# Prognostic factors in primary adenocarcinoma of the small intestine: 13-year single institution experience

**DOI:** 10.1186/1477-7819-6-12

**Published:** 2008-01-31

**Authors:** Kongkrit Chaiyasate, Akhilesh K Jain, Laurence Y Cheung, Michael J Jacobs, Vijay K Mittal

**Affiliations:** 1 Department of Surgery, Providence Hospital and Medical Centers, Southfield, Michigan 48075, USA

## Abstract

**Background:**

Adenocarcinoma of the small bowel is a relatively rare malignancy as compared to the other malignancies of the gastrointestinal tract. Nonspecific presentation and infrequent occurrence often leads to a delay in diagnosis and consequent poor prognosis. Various other factors are of prognostic importance while managing these tumors.

**Methods:**

The medical records of a total of 27 patients treated for adenocarcinoma of the small bowel at Providence Hospital and Medical Centers from year 1990 through 2003 were reviewed retrospectively. Data were analyzed using SPSS software (version 10.0; SPSS, Inc., Chicago, IL). Survival analyses were calculated using the Kaplan Meier method with the log rank test to assess the statistical significance. The socio-demographics (age, gender) were calculated using frequency analyses.

**Results:**

The patients included nine males and eighteen females with a median age at diagnosis of 62 years. Only 48% of the patients had an accurate preoperative diagnosis while another 33% had a diagnosis suspicious of small bowel malignancy. None of the patients presented in stage 1. The cumulative five-year survival was 30% while the median survival was 3.3 years. There was no 30-day mortality in the postoperative period in our series.

**Conclusion:**

The univariate analysis demonstrated that tumor grade, stage at presentation, lymph nodal metastasis and resection margins were significant predictors of survival.

## Background

Although the small bowel represents 90% of the surface area and 75% of the length of the alimentary tract and is located between two organs with high cancer incidence (i.e., stomach and colon), malignant neoplasm of the small bowel fall in the category of rare neoplasms. They account for only 2% of all GI malignancies. Even though, the first collective series of malignant small bowel neoplasm was published by Leichtenstein [1] in 1876, small bowel neoplasms continue to present a challenge to the clinician due to their infrequency, nonspecific symptoms and a delay in diagnosis. While the projected incidence in the United States is 22,280 cases of gastric cancer and 148,610 cases of colorectal cancer for the year 2006, the similar figure for small bowel cancer is only about 6,170 cases [2]. The prognosis of primary small bowel cancer remains dismal with 5-year survival rates ranging from 20% to 30%. This is a retrospective study aimed to report our experience with diagnosis and management of adenocarcinoma of the small bowel over the last decade in an effort to determine the factors influencing the long-term survival. The median follow up of patients was 7.9 years ranging from 0.6 to 13 years.

## Patients and Methods

This study includes a total of 27 patients who were diagnosed with adenocarcinoma of the small bowel at Providence Hospital between 1990 and 2003. The primary tumor was located between the duodenum and the ileum. However, periampullary cancers and patients with competing malignancies were excluded from this analysis. Data on demographics, presenting symptoms, diagnostic methods, surgical procedures, histopathology and the outcome of the patients were abstracted retrospectively from the medical records and tumor registry. The follow-up ranged from 0.6 to 13 years, median follow up being 7 years. The TNM categories and the extent of residual tumor after resection were classified according to the UICC 1997 criteria. Data were analyzed using SPSS software (version 10.0; SPSS, Inc., Chicago, IL). Survival analyses were calculated using the Kaplan Meier method with the log rank test to assess the statistical significance. The socio-demographics (age, gender) were calculated using frequency analyses. A p-value of < 0.05 was used to indicate statistical significance.

## Results

### Age/sex/symptoms

Of the 27 patients, nine were male and 18 were female. The median age at the time of diagnosis was 62 years. The majority (62.9%) of the patients were between 61–70 years of age and 35% were between 50–60 years old. The initial symptoms and physical signs are shown in Table 1.

**Table 1:** Initial symptoms of the patients

	N (%)
Nausea/vomiting	20 (74%)
Abdominal pain	17 (63%)
Melena	13 (48%)
Weight loss	10 (37%)
Anemia	9 (33%)
Palpable abdominal mass	9 (33%)
Dyspepsia complaints	9 (33%)
Intestinal obstruction	6 (22%)
Jaundice	4 (15%)

The diagnostic methods used are shown in Table 2. Thirteen of 27 patients (48%) were operated on with a proven diagnosis, an additional nine patients (33%) had a diagnostic suspicion of a small bowel tumor, and five patients (19%) had an unclear preoperative diagnosis. Interestingly, all the patients with duodenal adenocarcinoma (13 out of 27 patients) had a confirmed preoperative diagnosis. For the jejunal and ileal adenocarcinomas, the uncertainty in preoperative diagnosis was encountered irrespective of the duration of the symptoms, the location of the tumor within the small bowel or the diagnostic procedures used. The mean time to establish diagnosis is 14 days. None of the patients in our study had a known diagnosis of either celiac disease or Crohn's disease.

**Table 2:** Diagnostic methods applied to the patients

	**N (%)**
Abdominal ultrasonography	25 (93%)
Laparotomy	14 (52%)
Abdominal computed tomography	27 (100%)
Contrast radiography of small bowel	11 (40%)
Contrast radiography of stomach and duodenum	2 (7%)
Gastroduodenoscopy	13 (48%)
Mesenteric Angiography	5 (18%)
Tagged-RBC scan	4 (15%)

### Location of tumor/operative procedures/morbidity/mortality

The tumor was located in the duodenum in 48% of the patients while 22% had a lesion in the jejunum and 30% had a lesion in the ileum. Elective surgery was performed in 89% and emergency surgery in 11% of the patients. The operative procedures performed are listed in Table 3.

**Table 3:** Operative procedures

**Operative Procedure**	**N (%)**
Palliative bypass	3 (15%)
Pancreaticodoudenectomy	8 (30%)
Segmental resection with primary anastomosis	7 (26%)
*En bloc *resection	9 (33%)

Two patients with jejunoileal tumors undergoing emergency procedures were found to have superficial liver nodules, and one patient with similar pathology undergoing elective procedure was found to have superficial liver nodules and a distal pancreatic mass. The liver nodules and distal pancreatic mass in the latter patient were resected in the same sitting. For the duodenal adenocarcinomas associated with liver metastases, surgical bypass was all that was performed to palliate the obstructive symptoms. For all jejunoileal resectable tumors, systemic lymph node dissection was carried out which entailed a resection extending into the base of mesentery of the diseased segment of the small bowel.

Overall eight patients (30%) had postoperative complications: three wound infections (two in duodenal tumors and one in jejunal tumor), three pancreatic fistulas, one case of pneumonia (duodenal tumor), and one anastamotic leakage (duodenal tumor). All three patients with pancreatic fistulas were managed successfully with conservative management only and did not require surgical intervention. The complications encountered are depicted in Table 4. No 30-day mortality in this series was noted.

**Table 4:** Complications

**Complication**	**N (%)**
Wound infection	3(11%)
Pancreatic fistula	3(11%)
Pneumonia	1(4%)
Anastamotic leak	1(4%)

### Survival statistics

**Figure 1:**
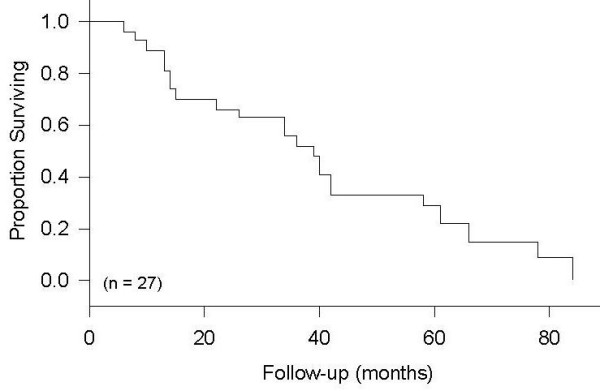
Overall Survival.

**Figure 2:**
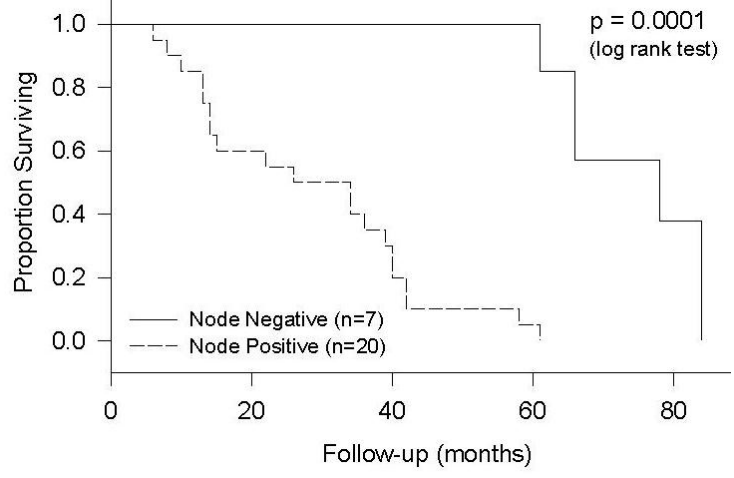
Survival by Lymph Node Involvement.

The median survival of the patients included in this study was 39 months (Figure 1). The overall five-year survival was 30%. Using Kaplan-Meier statistics, there was no detected influence of age and gender on the survival of the patients. However, several other prognostic factors were found influencing the survival of these patients. The median survival was 66 months for patients with well-differentiated tumors; 40 months with moderately differentiated tumors and 14 months with poor differentiation. These differences were statistically significant (p < 0.0001). The existence of distant metastasis was also found to be significant predictor of survival, (p < 0.0001). Nodal negative patients were found to have a median survival of 78 months, whereas, nodal positive patients had a median survival of 26 months. This difference in survival was found to be statistically significant (p < 0.0001, Figure 2).

**Figure 3:**
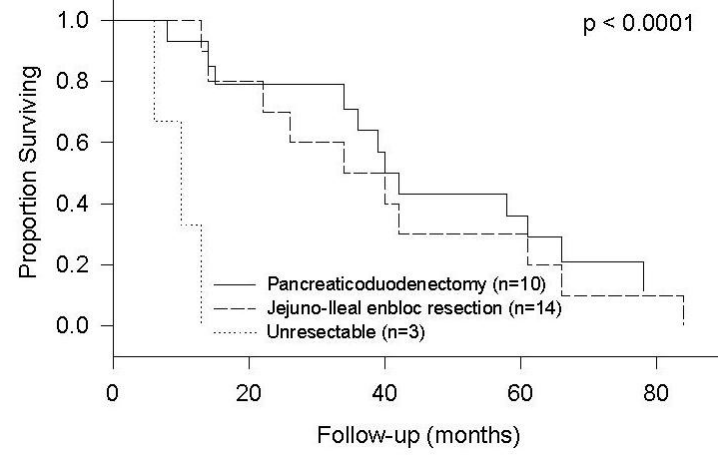
Survival by Resection Procedure.

The difference in survival of patients with resected jejunoileal adenocarcinomas (including radical *en bloc *resection and metastasectomy) and those with pancreaticoduodenectomy for duodenal adenocarcinomas was not statistically significant even though there was a trend towards better survival for patients undergoing enbloc resection for jejunoileal tumors (p = 0.59, Figure 3). However, median survival of patients with unresectable tumor was only 10 months, and the difference was statistically significant from those with a resectable tumor (p < 0.0001, Figure 3). The difference in survival of patients undergoing radical *en bloc *resection for jejunoileal tumors as compared to those undergoing pancreaticoduodenectomy was, however, not statistically significant.

**Figure 4:**
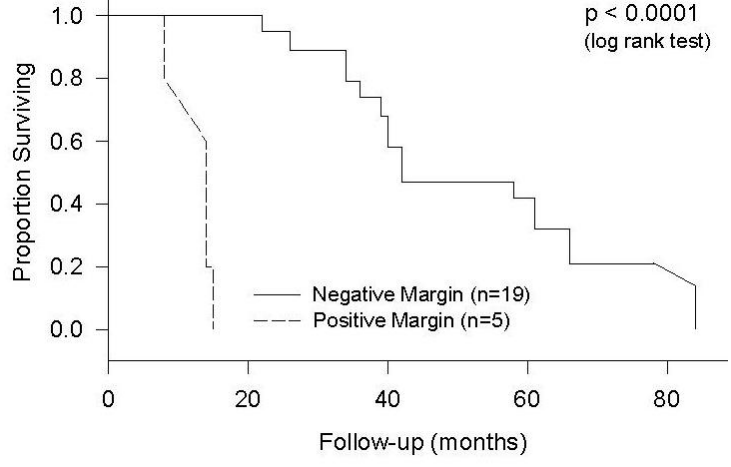
Survival by Margin Involvement*. 3 patients had unresectable tumors, and, therefore, have been excluded from this analysis.

In the current study, two patients with resected duodenal tumors had a positive microscopic margin (R1) on permanent section, and three patients with jejunoileal tumors had gross residual tumor after undergoing metastectomy (R2). Median survival was 14 months for those with a residual tumor (R1 and R2 positive margin), and 42 months for those with R0 or negative margin (p < 0.0001, Figure 4).

**Figure 5:**
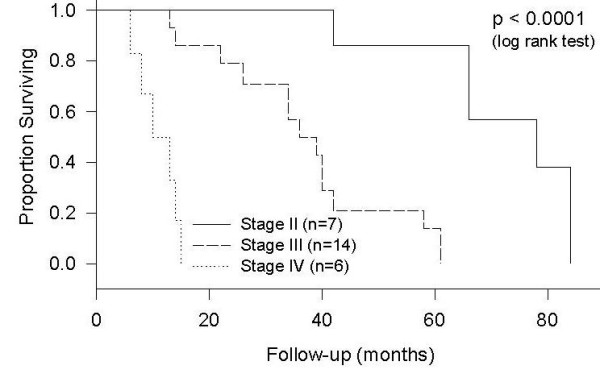
Survival by Stage.

**Figure 6:**
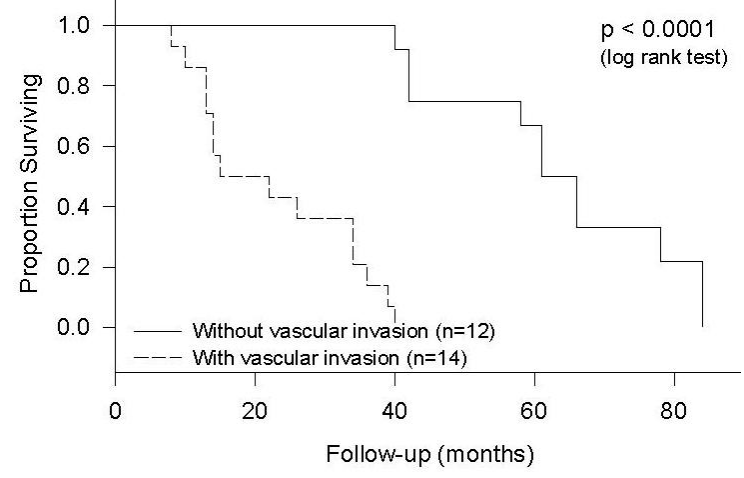
Survival by Vascular Involvement*. One out of 27 patients did not have data on vascular invasion in the histopathological report.

Another significant prognostic factor of survival was the stage of tumor at the time of diagnosis. Patients with stage IV tumors had a median survival of ten months, those with stage 3 tumors had a median survival of 36 months and those with stage II tumors had a median survival of 78 months (p < 0.0001, Figure 5). None of the patients had a stage I tumor at diagnosis. Vascular invasion also showed a significant difference in survival (p < 0.0001, Figure 6). Patients with vascular invasion had a median survival of 15 months and those without vascular invasion had a median survival of 61 months. Even though the survival of patients with tumor located in the duodenum tended to be lower than that for tumors located distally, the location of tumor within the small bowel was not found to be a statistically significant prognostic factor. Five patients with stage III adenocarcinoma and four patients with stage IV adenocarcinoma received chemotherapy. The results of this study showed no significant difference between the survival rates of patients who received chemotherapy as compared to those who did not.

## Discussion

Primary malignant tumors of the small bowel are rare. These are mainly adenocarcinomas followed in decreasing order by carcinoid tumors, non-Hodgkin lymphomas, gastrointestinal stromal tumors, melanomas, and other rare entities. In the United States, the incidence of all types of small bowel cancer is estimated to be approximately 5,300 cases per year and approximately 1,100 patients die from small bowel cancer each year. The development of an adenocarcinoma of the small bowel has been related to the mucosal contact time with bile acid solutions. Ross et al. [3] showed that the frequency of tumor distribution within the small bowel correlates with the length of mucosal contact with pancreatico-biliary secretions, implicating bile as a possible carcinogen. This is supported by findings that the active and passive transport of bile acid solutions is limited to the ileum [4].

In concordance with other reports, adenocarcinomas located in the small bowel, as other malignant entities of the small bowel, are observed mainly between 50 and 70 years of age [5-14]. In general, an accurate preoperative diagnosis has been reported only in 30%–72% of cases [10,11,15-24]. The clinical signs and symptoms may vary with the tumor site, size, and existence of ulceration. The common presenting signs and symptoms in our series were nausea, vomiting, abdominal pain, melena, weight loss, anemia, and a palpable mass, none of which was pathognomonic for small bowel tumors. All duodenal adenocarcinomas were diagnosed preoperatively by a gastro-duodenoscopy. For tumors in the jejunum and ileum, computer tomography and small bowel contrast study provided clues suggestive of small bowel tumor. Upper gastrointestinal tract series with small intestinal follow through is one of the most useful diagnostic tests. It yields an accurate diagnosis in 50 to 70% of patients with the neoplasm of small intestine [23]. Localization of intermittent-bleeding small bowel tumors through angiography and tagged-red blood cell radioisotope scan was also helpful in our study. Depending on the clinical symptoms, an emergency operation may be necessary. Seven patients (26%) in this study received emergency surgical treatment. Three of them had gastrointestinal hemorrhage and five had intestinal obstruction. Of importance is the fact that all the tumors requiring emergency surgery were located in the jejunum or the ileum. Thus, an accurate pathologic diagnosis could be achieved intraoperatively in these cases. The rate of diagnosis of small bowel tumors of all types by laparotomy varies between 40 and 80% in the literature [25]. In our study, the rate of diagnosis during laparotomy for small bowel adenocarcinoma was 52%. As known from the literature [14,24], adenocarcinomas are predominant in the duodenum. The more distal tumors were found more frequently in the jejunum than the ileum, which, however, is not a case in our study (30% in the ileum, and 22% in the jejunum). Brucher *et al*., found no patient with adenocarcinoma of the ileum in their series [14]. Recently, Dabaja *et al*., reported a 13% incidence of adenocarcinoma in the ileum [24].

In 1990 Sellener described an adenoma-adenocarcinoma-sequence [26] and In 1992 Lashner reported Crohn's disease as a risk factor in developing adenocarcinomas in the small bowel [27]. Rodriguez-Bigas *et al *[28], found an association between hereditary nonpolyposis colorectal carcinoma (HNPCC) patients and the increased risk of small bowel adenocarcinoma. In a review by Groves *et al*. [29], a total of six out of 114 patients of familial adenomatous polyposis (FAP) developed duodenal adenocarcinoma over a follow-up period of ten years. None of the patients in our study were known to have FAP, Crohn's disease or HNPCC.

The type of surgery varied according to the operating surgeons. For duodenal adenocarcinomas, 62% (eight out of 13 patients) of patients underwent pancreaticoduodenectomy and 15% (two out of 13 patients) underwent segmental duodenal resections with curative intent. Palliative bypass procedures were performed for the remaining patients with metastatic adenocarcinomas of the duodenum. For adenocarcinomas of the jejunum and ileum, Nine out of 14 patients underwent *en bloc *radical resection, which included three patients with metastectomy, and five out of 14 patients with localized diseases underwent segmental resections. When performing analysis, the palliative procedures had the shortest median survival (10 months) when compared to pancreaticoduodenectomies (34 months) and radical resections of the jejunoileal diseases (40 months). The demand of a higher technical expertise for resection of duodenal tumors as compared to resectable jejunoileal tumors may explain the inferior survival of patients with duodenal tumors, as demonstrated by the fact that significant morbidity in our series occurred only in patients with the tumor located in the duodenum. Importantly, every effort should be done to obtain R0 resection when dealing with small bowel adenocarcinoma because of a significant survival advantage.

Howe et al., [5] reviewed 4,995 patients with small bowel adenocarcinoma from the National Cancer Data Base from 1985–1995 and found the following factors to correlate with survival: patient age, tumor site (favoring jejunum and ileum), clinical staging, and whether curative resection was performed. Bakaeen *et al*. [6] and Ryder *et al*. [7] found tumor size, histologic grade, nodal metastases, and positive surgical margin to be prognostic factors predicting survival of adenocarcinomas of the duodenum. Brucher *et al*. [14] identified the presence of the residual tumor, tumor stage, lymph node metastasis, distant metastasis, lymphangiosis carcinomatosa, and vascular invasion as prognostic factors. Dabala *et al*. [24] recently reported that only cancer-directed surgery and lymph node involvement ratio to be independent predictors of overall survival in a multivariate analysis.

In our current study, the five-year survival was 30%, which is similar to that reported in pat literature [18]. We also found the presence of a positive node (p < 0. 0001), vascular invasion (p < 0.0001), and poor cellular differentiation (p < 0.0001) to be prognostic indicators, which is also analogous to the report of Brucher *et al *[14].

## Conclusion

A complete tumor resection has to be the aim of any curative surgical approach in patients with adenocarcinoma of the small bowel. The first step in improving the prognosis is to have an aggressive diagnostic approach in patients with unclear abdominal symptoms. The delay of diagnosis is responsible for the presentation of these patients at advanced tumor stages. Based on our data, the standard of the oncological surgery should be a systemic lymph node dissection and a radical *enbloc *R0 resection. Moreover, further investigation into the fundamental mechanisms driving the initiation and progression of small bowel cancers is needed. Although such investigations are considered to be low priority, given the low incidence of this cancer, findings in these studies may have important implications for more prevalent cancers.

## Competing interests

The author(s) declare that they have no competing interests.

## Authors' contributions

**KC **– Study concept and design, acquisition of data, drafting of manuscript, statistical analysis

**AKJ **– acquisition of data, analysis and interpretation, revision of manuscript for its intellectual content

**MJJ **– Analysis and interpretation of data, revision of manuscript for its intellectual content

**LYC **– Analysis and interpretation of data, revision of manuscript for its intellectual content

**VKM **– Study concept and design, revision of manuscript for its intellectual content

All authors read and approved the final manuscript for publication.
